# Tumor Electric Field Therapy Inhibits TGF‐β/C1R Signaling Axis‐Driven Epithelial‐Mesenchymal Transition in Glioblastoma

**DOI:** 10.1002/cns.70738

**Published:** 2026-01-05

**Authors:** Junyi Chen, Yuyang Liu, Qi Liu, Hongyu Liu, Cheng Sun, Xu Chen, Xinchen Zhao, Jinxin Lan, Yaping Feng, Lilin Qin, Jialin Liu, Ze Li, Ling Chen

**Affiliations:** ^1^ Medical School of Chinese PLA Beijing China; ^2^ Department of Neurosurgery Chinese PLA General Hospital Beijing China; ^3^ Department of Neurosurgery, The Second Affiliated Hospital Chongqing Medical University Chongqing China; ^4^ Department of Neurosurgery 920th Hospital of Joint Logistics Support Force Kunming China; ^5^ Graduate School Kunming Medical University Kunming China; ^6^ Faculty of Hepato‐Pancreato‐Biliary Surgery Chinese PLA General Hospital Beijing China; ^7^ School of Medicine Nankai University Tianjin China; ^8^ Chongqing University Fuling Hospital Chongqing China

**Keywords:** complement C1R, EMT, glioblastoma, mesenchymal subtype, TGF‐β signaling pathway, tumor electric field therapy

## Abstract

**Background:**

Glioblastoma (GBM) is one of the most aggressive and treatment‐resistant primary brain tumors, with the mesenchymal subtype exhibiting particularly poor prognosis. Tumor electric field therapy (TEFT) has emerged as a promising adjunctive treatment, but its underlying molecular mechanisms remain incompletely understood.

**Methods:**

GBM functional states were analyzed using CancerSEA datasets. GBM cell lines were treated with 200 kHz TEFT at 2.2 V/m for 72 h. C1R was knocked down using siRNA and shRNA. Cell morphology, migration, invasion, proliferation, and signaling pathways were assessed through various assays. Findings were validated in animal models and clinical specimens.

**Results:**

C1R was identified at the intersection of TEFT‐downregulated genes, poor prognosis markers, and functional state genes. C1R was significantly upregulated in mesenchymal GBM and strongly correlated with epithelial‐mesenchymal transition (EMT). Single‐cell RNA sequencing revealed C1R was predominantly expressed in MES‐like malignant cells with high EMT signature scores. TEFT treatment induced morphological changes from elongated spindle‐shaped to rounded epithelial‐like morphology, increased E‐cadherin expression, and decreased mesenchymal markers (N‐cadherin, Vimentin, YKL‐40). Mechanistically, TEFT suppressed the TGF‐β/SMAD2/3/STAT3 signaling pathway, downregulating C1R expression. C1R knockdown significantly reduced tumor growth in vivo, while exogenous TGF‐β restored C1R expression and reversed the mesenchymal phenotype in a dose‐ and time‐dependent manner.

**Conclusion:**

TEFT inhibits GBM progression by suppressing the TGF‐β/SMAD2/3/STAT3/C1R axis, thereby attenuating EMT and reducing tumor aggressiveness. These findings uncover a novel mechanism of TEFT and identify C1R as a potential biomarker and therapeutic target for GBM.

## Graphical abstract

Tumor electric field therapy (TEFT) inhibits glioblastoma progression by suppressing the TGF‐β/SMAD2/3/STAT3/C1R axis to reverse epithelial‐mesenchymal transition (EMT). C1R knockdown reduces tumor growth and mesenchymal characteristics. This study identifies C1R as a key molecular target of TEFT and a potential biomarker for the aggressive mesenchymal subtype of glioblastoma.

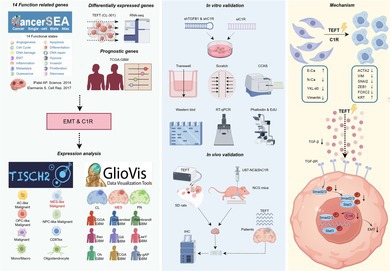

**Abbreviations**
CCK‐8: cell counting kit‐8
CGGA: Chinese Glioma Genome Atlas
DEGs: differentially expressed genes
DMEM: Dulbecco's modified Eagle medium
EdU: 5‐ethynyl‐2′‐deoxyuridine
EMT: epithelial‐mesenchymal transition
FBS: fetal bovine serum
GBM: glioblastoma
GTRD: Gene Transcription Regulation Database
HRQoL: health‐related quality of life
IHC: immunohistochemistry
MES: mesenchymal
MET: mesenchymal‐epithelial transition
NC: negative control
NCCN: National Comprehensive Cancer Network
PN: proneural
RPPA: reverse phase protein array
SD: standard deviation
ShRNA: short hairpin RNA
SiRNA: small interfering RNA
TCGA: The Cancer Genome Atlas
TEFT: tumor electric field therapy
TGF‐β: transforming growth factor‐beta
TISCH2: tumor immune single‐cell Hub 2
TME: tumor microenvironment
TMZ: temozolomide
UMAP: Uniform Manifold Approximation and Projection
WB: Western blot

## 1 Introduction

Glioblastoma (GBM) is the most common and aggressive primary brain tumor in adults, comprising approximately 15% of all intracranial tumors and 60%–75% of astrocytic tumors, with a 5‐year survival rate of only 6.9% [1, 2]. It is characterized by rapid growth, high invasiveness, and resistance to conventional treatments, making it one of the most challenging cancers to treat [3]. Despite advances in surgical resection, radiotherapy, and chemotherapy, the prognosis for GBM patients remains poor, with a median survival of only 15 months following diagnosis [4]. Recurrence is common, often occurring within months after treatment, and survival rates have seen limited improvement over the past few decades [5]. In recent years, tumor electric field therapy (TEFT) has emerged as a promising adjunctive treatment for GBM. TEFT uses low‐intensity, intermediate‐frequency electric fields to disrupt cell division, thereby inhibiting tumor growth and enhancing the effectiveness of other therapeutic modalities, such as chemotherapy and radiation [6, 7]. TEFT is administered as a postoperative adjuvant therapy following maximal safe resection and concurrent chemoradiotherapy, with continuous application of 200 kHz alternating electric fields for at least 18 h daily [8]. Clinical studies have demonstrated that TEFT, when combined with temozolomide (TMZ), significantly extends overall survival (OS) and progression‐free survival (PFS) in GBM patients [9, 10]. However, the underlying molecular mechanisms by which TEFT exerts its anti‐GBM effects remain incompletely understood.

GBM exhibits marked heterogeneity, with transcriptional profiling revealing distinct molecular subtypes, including proneural, neural, classical, and mesenchymal (MES) [11]. Among these, the MES subtype is particularly associated with the most aggressive behavior and the poorest prognosis [12]. MES tumors exhibit enhanced invasiveness, increased resistance to standard therapies like radiation and chemotherapy, and a tendency to relapse quickly after treatment. Wang et al. reported that in a cohort of 54 GBM patients, the MES subtype demonstrated inferior outcomes in both primary and recurrent settings compared to non‐MES tumors (primary: OS 15.2 vs. 17.8 months, PFS 6.1 vs. 7.1 months; recurrent: OS 15.3 vs. 18.5 months, PFS 4.1 vs. 8.7 months) [13]. Similarly, Wu et al. demonstrated that GBM patients with MES subtype had significantly shorter OS (11.7 months) compared to those with PN subtype (14.4 months), and MES clones exhibited greater resistance to radiotherapy (median survival fraction: MES, 0.60; PN, 0.47) [14]. The MES subtype is associated with the upregulation of genes involved in inflammation, extracellular matrix remodeling, and cell motility, contributing to the tumor's aggressive spread and therapeutic resistance [15, 16]. One of the key mechanisms underlying the aggressive nature of MES GBM is the activation of epithelial‐to‐mesenchymal transition (EMT), a process through which epithelial cells lose their adhesive properties and acquire mesenchymal traits with stronger migration and invasion capabilities [17, 18]. EMT plays a crucial role in GBM progression, facilitating tumor dissemination and local recurrence, which are major contributors to poor survival rates. Furthermore, EMT is closely linked to the resistance of GBM to various treatments, as it alters the tumor microenvironment (TME) and diminishes the efficacy of conventional therapies [19, 20].

Transforming growth factor‐beta (TGF‐β) is a critical mediator of EMT in GBM, promoting cellular changes that drive migration, invasion, and immune evasion [19, 21]. TGF‐β exerts its effects by activating both canonical (Smad‐dependent) and noncanonical (Smad‐independent) signaling pathways [22, 23]. The latest research showed that Enhydrin inhibits the EMT process of GBM through the Jun/Smad7/TGF‐β1 signaling pathway, leading to a significant decrease in cell proliferation, invasion, and migration. Multiple studies have shown TGF‐β signaling not only accelerates EMT but also contributes to a tumor‐supportive microenvironment that shields tumor cells from therapeutic interventions [24, 25, 26]. Understanding the complicated molecular mechanisms that regulate EMT, especially through TGF‐β signaling, is essential for developing new therapeutic strategies aimed at reversing EMT and overcoming resistance, ultimately improving the prognosis for GBM patients.

Complement C1r (C1R) is a crucial component of the major histocompatibility complex (MHC) class I pathway, which plays a key role in immune surveillance and tumor immunity [27]. Some studies have suggested that C1R may influence immune evasion and tumor progression by modulating the TME [28, 29]. In various cancers, including GBM, C1R has been shown to impact the ability of tumor cells to escape immune detection, potentially contributing to the aggressive nature of the disease [29, 30, 31, 32]. While the primary function of C1R has been associated with immune response regulation, emerging research indicates that its involvement in tumor growth and progression may extend beyond immune modulation [33, 34, 35]. In particular, its potential role in regulating molecular mechanisms such as EMT and influencing tumor cell behavior in GBM remains largely unexplored. Investigating how C1R affects GBM biology could uncover new therapeutic strategies and improve the efficacy of existing treatments.

In our previous study, we found that TEFT inhibited tumor cell viability, proliferation, and invasion in vitro, with random‐sequence fields exhibiting superior effects compared to unidirectional fields [1, 36]. The safety and efficacy of TEFT were further validated in a rat model [37]. In this study, we elucidated a novel molecular mechanism of TEFT therapy for GBM, focusing on complement component C1R and its role in EMT. We demonstrated that TEFT suppressed the TGF‐β/SMAD2/3/STAT3 signaling pathway, which downregulated C1R expression and subsequently inhibited EMT in GBM cells. C1R silencing reduced the migration, invasion, and proliferation ability of GBM cells, while exogenous TGF‐β restored C1R expression and reversed the mesenchymal‐to‐epithelial transition induced by C1R knockdown. Our findings uncover a novel mechanism of TEFT therapy for GBM, and C1R may serve as a promising biomarker and potential target for combination therapeutic strategies in GBM treatment.

## 2 Method

### 2.1 Cultures of GBM Cell Line

The U87 and U251 cell lines were obtained from the Institute of Basic Medicine at China Medical College. The cells were cultured in Dulbecco's Modified Eagle Medium high‐glucose (DMEM; Gibco) supplemented with 10% fetal bovine serum (FBS; Gibco). Cell culture was maintained in an incubator at 37°C with 5% CO_2_.

### 2.2 Tumor Electric Field Treatment on Cells

GBM cell line tumor electric field treatment referred to our previous research [1]. The clinically approved TEFT specifications for glioblastoma treatment operate within a frequency range of 100–300 kHz and field intensity of 1–3 V/cm [38]. Our previous systematic investigation within this therapeutic range demonstrated that 200 kHz frequency combined with 2.2 V/cm field strength yielded optimal antiproliferative effects against various glioblastoma cells [36]. Cells were seeded on 20‐mm glass slides (Nest 801008) at a density of 2 × 10^5^ cells/mL, with 150 μL of suspension per slide. After overnight incubation for cell adhesion, electric field treatment was applied using a TEFT device (CL‐301A) with a 200 kHz frequency and 2.2 V/cm field strength for 72 h. Control groups were maintained under identical conditions without TEFT exposure.

### 2.3 Clinical and Animal Sample Collection for TEFT

The study was approved by the Institutional Review Board of PLA General Hospital, with approval number S2018‐089‐01. Informed consent was obtained from all participating patients. Three paired paraffin‐embedded GBM tissue samples were collected from patients who underwent surgical resection both before and after TEFT. Patient selection criteria included histopathologically confirmed GBM diagnosis, completion of standard radiotherapy and temozolomide chemotherapy, and availability of adequate tumor tissue from both time points. TEFT was administered as alternating electric fields at 200 kHz with continuous daily application for ≥ 18 h/day [9, 39]. All tissue samples were processed using standard histological protocols and prepared for immunohistochemical staining. Additionally, GBM samples from rats subjected to TEFT were obtained from our previous study for further analysis [37].

### 2.4 GBM Related 14 Functional States Analysis via Cancer SEA Datasets

To identify genes associated with the 14 functional states in glioblastoma (GBM), we utilized single‐cell datasets related to GBM available in the CancerSEA database (http://biocc.hrbmu.edu.cn/CancerSEA/) [40]. The datasets selected for this analysis were from Darmanis et al., published in *Cell Reports* (2017) [41], and Patel et al., published in *Science* (2014) [42], both focused on brain tissue. Genes positively correlated with each of the 14 functional states (significantly promoting these functions) were identified and defined as “14 Functional States Genes” for subsequent analysis. The relationships between key genes and functional states were visualized using a correlation bubble plot. The size of the bubble represents the strength of the correlation.

### 2.5 Analysis of the Hub Gene Related to TEFT Anti‐GBM Effect

To identify GBM prognosis‐associated genes, RNA‐seq data from the TCGA‐GBM dataset was downloaded and processed from the TCGA database (https://portal.gdc.cancer.gov). Data corresponding to the TCGA‐GBM project processed via the STAR pipeline was extracted in TPM format along with clinical data. Data processing was performed using the formula log_2_ (value + 1). Prognosis‐associated genes were identified using R (version 4.2.1) and the survival package (version 3.3.1). Statistical analysis was conducted using Cox regression with *p* value adjustment method set as “none,” grouping based on survival percentiles (0%–50% vs. 50%–100%), and OS as the prognosis type. Genes with *p* < 0.05 and hazard ratio (HR) ≠ 1 were retained for further analysis. To identify TEFT anti‐GBM effector genes, differentially expressed genes (DEGs) analysis was performed based on our previous study [1]. DEGs were defined as genes with an absolute log_2_ fold change > 2 and adjusted *p* < 0.05. Genes with log_2_ fold change > 2 were classified as upregulated DEGs (indicating increased expression), while those with log_2_ fold change < 2 were classified as downregulated DEGs (indicating decreased expression). Genes without a corresponding gene name were excluded. DEGs meeting these criteria were retained for further analysis. Intersection genes between prognosis‐associated genes, TEFT anti‐GBM effector genes, and 14 functional states genes were identified and visualized using an UpSet plot.

### 2.6 Expression and Survival Analysis of C1R in Multiple GBM Datasets

Gene expression and matched clinical data for GBM and glioma samples were obtained from the GlioVis portal (http://gliovis.bioinfo.cnio.es/). Survival analyses were conducted using datasets including CGGA‐GBM, Gravendeel‐GBM, LeeY‐GBM, Phillips‐GBM, and Rembrandt‐GBM. Expression analyses were conducted using datasets including CGGA, Gravendeel, Rembrandt, TCGA, CGGA‐GBM, Gravendeel‐GBM, Rembrandt‐GBM, TCGA‐GBM (RNA‐seq), TCGA‐GBM (HG‐U133A), Bao‐GBM, Gill‐GBM, LeeY‐GBM, Oh‐GBM, Ivy‐GAP. Reverse phase protein array (RPPA) data from the TCGA‐GBM dataset (HG‐U133A) were analyzed to compare protein expression patterns between C1R‐high and C1R‐low groups, defined by the median mRNA expression level of C1R proteins, with significant differences (adjusted *p* < 0.05) visualized in a heatmap to highlight molecular distinctions. Single‐cell RNA sequencing data for GBM were obtained from the Tumor Immune Single‐cell Hub 2 (TISCH2) database (http://tisch.comp‐genomics.org/), using the GSE1311928 (10×) dataset to analyze the expression patterns of C1R gene in various GBM cell subpopulations. The distribution of HALLMARK EMT scores was analyzed on single‐cell Uniform Manifold Approximation and Projection (UMAP) plots, using the built‐in Single‐cell Signature Explorer tool available in the TISCH2 database. Cell populations with high EMT scores were identified to evaluate their functional roles in GBM.

### 2.7 Identification of Upstream Transcriptional Factors

To identify potential upstream transcriptional regulators of C1R, we performed a comprehensive analysis using the TF Target Finder platform (https://jingle.shinyapps.io/TF_Target_Finder/). The analysis incorporated six prominent transcription factor databases: ChIP‐Atlas, GTRD, CHEA, ENCODE, KnockTF, and hTFtarget. A Log_2_ FC Threshold of 0.5 was applied to filter relevant transcription factors associated with C1R expression. The intersection of predictions from all six databases was used to identify common transcription factors, ensuring a comprehensive and unbiased approach to uncovering potential regulatory elements associated with C1R expression.

### 2.8 Proteogenomics Analysis of C1R

To illustrate the expression patterns and potential regulatory mechanisms of C1R protein, the LinkedOmicsKB database (https://kb.linkedomics.org/) was used to conduct analyses including cis‐association, gene set enrichment analysis (GSEA), and phenotype and mutation association analyses on pan‐cancer and GBM levels. In the cis‐association section, associations between omics data (protein, mRNA, SCNV, and methylation data) of C1R at the pan‐cancer and GBM levels were explored to detect potential regulatory mechanisms of C1R protein. In the phenotype and mutation association section, correlation analyses between levels of EMT, TGF‐β signaling, various scores, immune cell types, and C1R levels (protein and mRNA) were operated. Furthermore, GSEA on C1R protein was constructed on GBM and pan‐cancer levels.

### 2.9 Transcriptional Sequencing of U87 Cells

RNA sequencing (RNA‐seq) analysis was conducted on U87 cells divided into ShC1R (C1R knockdown) and NC (control) groups. Total RNA extraction was performed using Trizol reagent (ThermoFisher) according to the manufacturer's instructions. Oligo(dT) beads were utilized to isolate poly(A) RNAs and ncRNAs from the extracted total RNA. The enriched RNA samples were used to construct sequencing libraries, which were subsequently sequenced on an Illumina platform by Tsingke Corporation. Data preprocessing involved adapter trimming and removal of low‐quality/complexity reads. Cleaned sequences were mapped to the GRCh38.p13 human reference genome with HISAT2. All sequencing data, including both raw and processed files, have been submitted in the NCBI Gene Expression Omnibus (GEO) database.

### 2.10 Phalloidin Staining

Phalloidin staining was performed to analyze cellular morphological changes by visualizing the actin cytoskeleton. According to the manufacturer's instructions, cells were cultured on laser confocal culture dishes (Nest, cat. no. 801001) and fixed with 4% paraformaldehyde for 15 min at room temperature, followed by permeabilization with 0.5% Triton X‐100 for 5 min. Subsequently, cells were incubated with phalloidin‐conjugated fluorophore (Beyotime, 1:100 dilution) for 30 min in the dark and counterstained with DAPI (1 μg/mL) for 5 min to label nuclei. Fluorescence images were acquired using a confocal microscope (Olympus, FV1000), with representative fields captured for analysis.

### 2.11 Quantitative RT‐PCR

Total RNA was extracted from cell samples using the RNeasy Mini Kit (Qiagen). cDNA synthesis was performed with SuperScript III Reverse Transcriptase (Invitrogen) and random hexamer primers. Quantitative real‐time PCR was carried out on the Applied Biosystems 7500 Real‐Time PCR System under the following conditions: 95°C for 10 min, then 40 cycles of 95°C for 15 s and 60°C for 1 min. Each reaction was run in triplicate, using β‐actin as the reference gene. Relative target gene expression was calculated via the $2^{-\Delta \Delta C_{t}}$ method. Primer sequences used can be found in Table S1.

### 2.12 Transient Transfection and Lentiviral Transfection

For transient transfection and lentiviral transfection of the C1R gene, specific siRNA targeting C1R for transient transfection and shRNA for lentiviral transfection were designed and synthesized by Tsingke Biotechnology Company (Beijing). For transient transfection, cells were seeded in six‐well plates to achieve 70%–90% confluency on the day of transfection. Transfection was performed using Lipofectamine 2000 transfection reagent (Invitrogen, 11668‐019) according to the manufacturer's protocol. Cells were incubated for 48 h post‐transfection, and the knockdown efficiency was evaluated by qRT‐PCR and Western blot analysis. For lentiviral transfection, shRNA sequences targeting C1R were cloned into a lentiviral vector plasmid, and HEK293T cells were transfected using Lipofectamine 2000 to generate lentiviral particles. The virus‐containing supernatant was collected and used to infect the target cells. After 24 h, the medium was replaced with medium containing 2 μg/mL puromycin for selection. The infected cells were cultured for 2–3 weeks until single‐cell colonies appeared. Stable knockdown clones were expanded, and knockdown efficiency was confirmed by RT‐qPCR and Western blot. The siRNA and shRNA sequences used can be found in Tables S2 and S3.

### 2.13 Wound Healing and Transwell Assay

Cells were seeded into 6‐well plates and cultured to approximately 90% confluence. A sterile 200 μL pipette tip was used to create a linear scratch wound, and the cells were washed twice with PBS to remove debris before being cultured in serum‐free DMEM. Wound healing was monitored at 0 and 24 h using an inverted microscope, and the migration distance was measured and analyzed using ImageJ software. For the transwell migration assay, 1 × 10^4^ cells were resuspended in serum‐free medium and seeded in the upper chamber of transwell inserts with an 8 μm pore size (Corning, 3422). The lower chamber was filled with 600 μL of DMEM containing 10% FBS as a chemoattractant. After 18 h of incubation at 37°C in 5% CO_2_, non‐migrated cells on the upper surface of the membrane were gently removed with a cotton swab. Migrated cells on the lower surface were fixed with 4% paraformaldehyde for 15 min, stained with 0.1% crystal violet for 20 min, and counted under an inverted microscope (Olympus BX51, Japan) in three randomly selected fields. For the transwell invasion assay, the procedure was identical, except the transwell inserts were precoated with Matrigel (Corning, 356234) to mimic the extracellular matrix. All experiments were performed in triplicate.

### 2.14 Clone Formation and Cell Counting Kit‐8 (CCK‐8) Assay

Clone formation and cell proliferation assays were performed to evaluate the clonogenic ability and growth potential of cells. For the clone formation assay, cells were seeded in 6‐well plates at a low density (1000 cells/well) and cultured for 14 days. Colonies were fixed with 4% paraformaldehyde, stained with crystal violet, and counted manually. The clone formation rate was calculated as the number of colonies divided by the number of seeded cells. For the cell proliferation assay, the Cell Counting Kit‐8 (Solarbio, CA1210) was used according to the manufacturer's instructions. Cells were seeded in 96‐well plates at a density of 3000 cells/well and cultured for 24, 48, 72, and 96 h. Absorbance was measured at 450 nm using a microplate reader to assess cell viability and proliferation.

### 2.15 EdU Proliferation Assay

Cells were seeded on laser confocal culture dishes (Nest, cat. no. 801001) and cultured under standard conditions. EdU incorporation was performed using the EdU Cell Proliferation Detection Kit (GeneAdv, GAS1080) following the manufacturer's instructions. EdU was added to the culture medium at a final concentration of 10 μM and incubated for 2 h at 37°C. Cells were then fixed with 4% paraformaldehyde, permeabilized with 0.5% Triton X‐100, and incubated with the reaction cocktail to label EdU‐positive cells. Nuclei were counterstained with DAPI, and images were captured using a confocal microscope (Olympus, FV1000). The percentage of EdU‐positive cells was quantified by ImageJ software.

### 2.16 Western Blot

Proteins were extracted from samples using RIPA lysis buffer (Beyotime, China) supplemented with protease inhibitor cocktail (MCE, China). The extracted proteins were separated by SDS‐PAGE on 4%–12% polyacrylamide gels and transferred onto polyvinylidene difluoride (PVDF) membranes. Membranes were blocked with 5% skim milk prepared in Tris‐buffered saline containing 0.1% Tween‐20 (TBST) for 1 h at room temperature, followed by incubation with primary antibodies (1:1000 dilution) specific to the target proteins overnight at 4°C. After thorough washing with TBST, membranes were incubated for 1 h at room temperature with horseradish peroxidase‐conjugated secondary antibodies (1:5000 dilution). Protein bands were visualized using enhanced chemiluminescence reagents, and band intensities were quantified using ImageJ software. Antibodies used can be found in Table S4.

### 2.17 Chromatin Immunoprecipitation (ChIP)

Cells were fixed with 1% formaldehyde in culture medium, and crosslinking was terminated by glycine treatment. Following harvest by scraping, cells were lysed in ChIP lysis buffer, and chromatin was fragmented to 150–900 bp using micrococcal nuclease digestion. After setting aside 2% of each sample as input control, the chromatin lysate was immunoprecipitated overnight at 4°C with the indicated antibodies or IgG control. Immune complexes were captured by incubation with Protein G Agarose Beads for 2 h at 4°C. Following elution and reverse crosslinking, DNA was purified and analyzed by RT‐qPCR using primers detailed in Table S1.

### 2.18 Immunohistochemistry (IHC)

The tissues were fixed in 4% paraformaldehyde, embedded in paraffin, and sectioned into 4 μm thick slices. Sections were mounted on slides and processed according to a previously described protocol [36]. Slides were incubated overnight at 4°C with a primary antibody diluted in 1% goat serum PBS solution, followed by washing and incubation at room temperature for 1 h with a secondary antibody. Staining was visualized using the ABC Horseradish Peroxidase kit with DAB as the chromogen, and nuclei were counterstained with hematoxylin. Staining patterns were independently evaluated by two pathologists, blinded to clinical and animal data. Positive staining was scored based on the percentage of stained cells (0: < 5%, 1: 5%–25%, 2: 26%–50%, 3: 51%–75%, 4: > 75%) and staining intensity (0: none, 1: light yellow, 2: yellowish brown, 3: brown). The final IHC score was calculated as the product of the two scores.

### 2.19 In Vivo Experiments

The xenograft tumor experiment in 8‐week‐old female NCG mice was conducted with approval from the Institutional Animal Care and Use Committee (IACUC) of GemPharmatech Co. Ltd. (Beijing, China) (Animal Protocol No. GPT‐BJAP20241021‐1). U87 cells, stably transfected and in the logarithmic growth phase, were prepared for transplantation. The cells were resuspended in PBS at a concentration of 2 × 10^6^ cells/100 μL. Subsequently, 100 μL of the cell suspension was subcutaneously injected into the axillary region of the mice. The mice were randomly divided into two groups: the NC group and the shC1R group, with six mice in each group. Following implantation, the mice were monitored every 2–3 days to assess their condition. Tumor growth was tracked by measuring the xenograft dimensions, and tumor volume was calculated using the formula: (longest diameter × shortest diameter^2^)/2. On Day 21 postimplantation, all mice were euthanized, and the tumors were excised and weighed.

### 2.20 Statistical Analysis

The Shapiro–Wilk test was performed to evaluate the normality of data distribution. Differences between two groups were analyzed using the Student's *t*‐test. One‐way analysis of variance (ANOVA) was applied for comparisons among three or more groups, followed by Tukey's post hoc test. Patients were categorized into high‐expression (50%–100%) and low‐expression (0%–50%) groups according to median mRNA expression levels. Survival analysis was performed using the survival R package (version 3.3.1), with the results visualized through the survminer package (version 0.4.9). Statistical analyses were conducted in R software (version 4.2.1), and a two‐sided *p*‐value of < 0.05 was deemed statistically significant.

## 3 Result

### 3.1 C1R Correlated With TEFT Anti‐Tumor Effects and EMT in GBM

**FIGURE 1:**
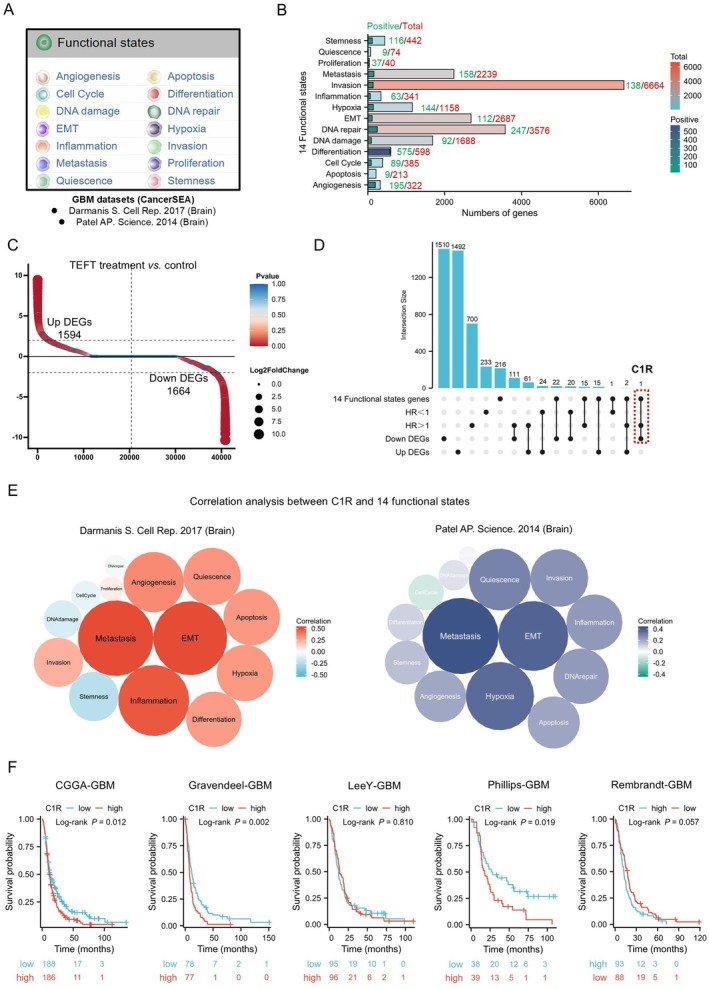
Identification of key functional states and genes involved in TEFT anti‐GBM. (A) Fourteen functional states relevant to tumor biology as defined in CancerSEA database. (B) Bar chart showed genes associated with each functional state, focusing on those with positive correlations. (C) DEGs of GBM cell lines following TEFT treatment versus control. (D) Upset plot identified C1R at the intersection of functional state genes, poor prognosis markers (HR > 1, *p* < 0.05), and TEFT‐downregulated genes (|log_2_ FC| > 2, *p* < 0.05). (E) Correlation analysis between C1R expression and functional states across two GBM datasets. (F) Survival analysis of multiple GBM datasets showed that high C1R expression correlates with poor prognosis.

To elucidate the functional impact of TEFT on GBM, we performed comprehensive analyses using two independent GBM datasets from the CancerSEA database to assess 14 key tumor functional states, including angiogenesis, apoptosis, cell cycle regulation, DNA damage repair, epithelial‐to‐mesenchymal transition, inflammation, and others (Figure 1A). Based on gene expression profiles from these datasets (Figure 1B), we identified candidate genes potentially linked to these functional states and focused on genes positively contributing to these processes.

We further analyzed transcriptomic data from GBM cell lines before and after TEFT treatment (Figure 1C), utilizing sequencing data from our previous research [1]. The results revealed that TEFT treatment led to the upregulation of 1594 genes (Up DEGs) and downregulation of 1664 genes (Down DEGs). To identify critical candidate genes, we employed an Upset plot for triple‐condition screening (Figure 1D), requiring genes to simultaneously meet the following criteria: (1) association with the 14 functional states; (2) correlation with poor prognosis (HR > 1); and (3) downregulation following TEFT treatment. This stringent filtering identified only C1R as meeting all conditions, suggesting that C1R may regulate GBM prognosis and treatment response by influencing these functional states.

Further correlation analysis between C1R and the 14 functional states in two GBM datasets showed that C1R was highly correlated with EMT and metastasis functional states in both datasets (Figure 1E). Given that GBM rarely metastasizes to distant sites [43], these findings suggest that C1R likely exerts its effects in GBM primarily through regulation of EMT functionality.

To further validate the association between C1R and EMT across multiple cancer types, we performed comprehensive multi‐omics analyses using pan‐cancer datasets. Pan‐cancer analysis revealed robust correlations between C1R abundance (at both protein and RNA levels) and EMT signatures across multiple cancer types, including BRCA, CCRCC, COAD, GBM, HNSCC, LSCC, LUAD, OV, PDAC, and UCEC (Figure S1A). Specifically, in GBM, C1R protein levels demonstrated a strong positive correlation with HALLMARK EMT scores (Rho = 0.66, *p* = 2.2e‐16) (Figure S1B), further supporting the critical role of C1R in EMT regulation. GSEA provided additional evidence for this relationship. In pan‐cancer cohorts, C1R protein expression was significantly enriched in the HALLMARK EMT pathway (size = 193, leading edge number = 123, ES = 0.80521, NES = 2.0027, *p* < 2e‐10) (Figure S1G). Notably, GSEA analysis in GBM specifically revealed even more pronounced enrichment of the HALLMARK EMT pathway associated with C1R expression (size = 173, leading edge number = 102, ES = 0.79659, NES = 2.4016, *p* < 2e‐10) (Figure S1H), indicating that the C1R‐EMT association is particularly prominent in GBM compared to other cancer types.

Finally, survival analyses across multiple independent GBM cohorts from the Gliovis platform confirmed that high C1R expression was significantly associated with poor prognosis in CGGA‐GBM (*p* = 0.012), Gravendeel‐GBM (*p* = 0.002), and Phillips‐GBM (*p* = 0.019) cohorts (Figure 1F). These results indicate that C1R may play a crucial role in TEFT treatment by influencing key GBM functions, particularly the EMT process.

### 3.2 C1R Was Highly Expressed in MES‐Like Malignant Cells and Mesenchymal Subtype GBM

**FIGURE 2:**
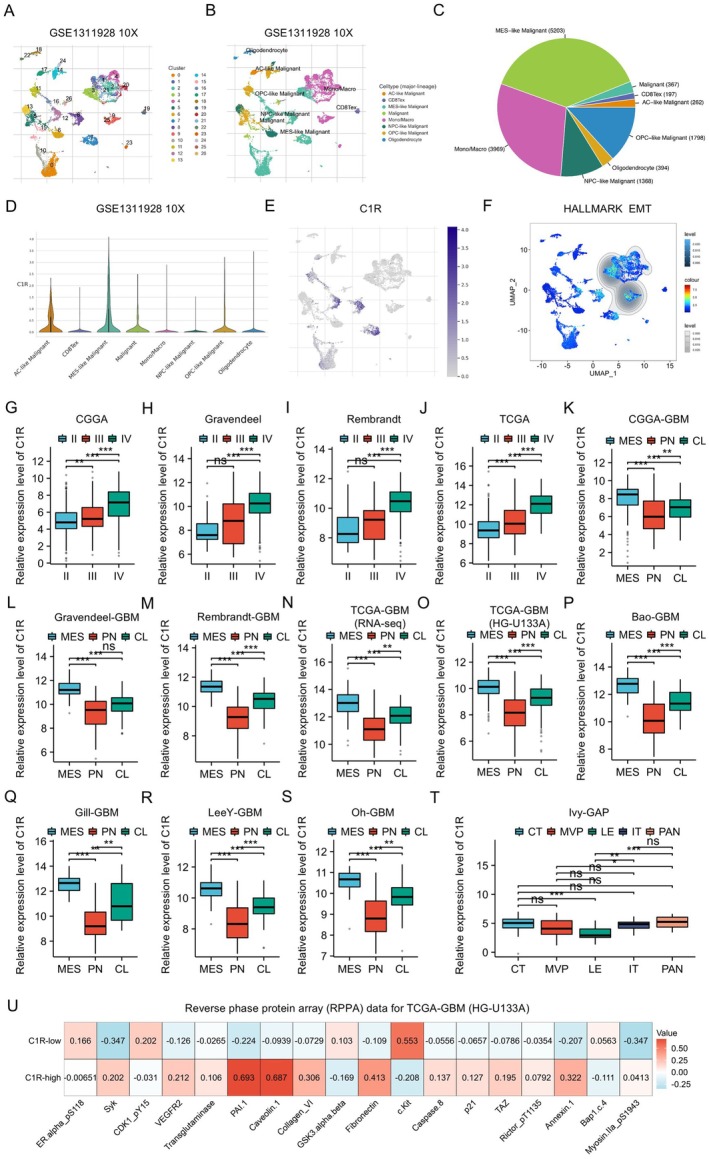
The expression pattern and prognostic value of C1R. (A, B) Analysis of single‐cell RNA‐seq data from the GSE1311928 dataset showed heterogeneous cell populations in GBM and annotation of major cell clusters. (C) Pie chart depicted the proportion of cell subtypes identified in the single‐cell analysis. (D) Violin plot demonstrated differential C1R expression across various cell clusters. (E) UMAP plot showed C1R was highly expressed in MES‐like malignant cells. (F) UMAP plot illustrated the distribution of HALLMARK EMT signature scores across cell clusters. Color intensity indicates the value of EMT scores, with deeper blue representing stronger EMT features. (G–J) Analysis of C1R expression in multiple glioma datasets with different grades of gliomas. (K–S) Comparative analysis of C1R expression across molecular subtypes of GBM: mesenchymal (MES), proneural (PN), and classical (CL) in nine independent cohorts. *p < 0.05, **p < 0.01, ***p < 0.001, ns = not significant, Kruskal–Wallis test and Dunn's post hoc test. (T) Distribution of C1R expression across distinct anatomical regions of GBM based on the Ivy GAP database. Cellular tumor (CT), pseudopalisading cells around necrosis (PAN), leading edge (LE), infiltrating tumor (IT), microvascular proliferation (MVP). (U) Heatmap of RPPA data from TCGA‐GBM (HG‐U133A) compared protein expression between C1R‐low and C1R‐high groups.

Single‐cell RNA sequencing analysis demonstrated heterogeneous expression patterns of C1R across GBM cells (Figure 2A). Using the TISCH2 database to analyze the GSE1311928 GBM cohort, we identified multiple cell subpopulations, including AC‐like malignant, CD8Tex, MES‐like malignant, malignant, mono/macro, NPC‐like malignant, OPC‐like malignant, and oligodendrocyte (Figure 2B,C). Violin plots revealed significantly elevated C1R expression in the MES‐like malignant cells (Figure 2D). Further UMAP visualization demonstrated that C1R was highly expressed in MES‐like malignant cells, showing a strong correlation with mesenchymal characteristics (Figure 2E). The cells with high C1R expression were enriched in regions showing elevated HALLMARK EMT signature scores, suggesting a close association between C1R and the epithelial‐mesenchymal transition process (Figure 2F).

At the glioma tissue level, we examined C1R expression patterns across multiple cohorts. Analyses of CGGA, Gravendeel, Rembrandt, and TCGA datasets consistently showed that C1R expression levels increased with glioma grade progression, with the highest expression in GBM (Figure 2G–J). Additionally, in the CGGA‐GBM cohort, C1R expression was significantly higher in the mesenchymal (MES) subtype compared to the proneural (PN) and classical (CL) subtypes (Figure 2K). This finding was validated across multiple independent GBM cohorts, including Gravendeel‐GBM, Rembrandt‐GBM, TCGA‐GBM (RNA‐seq), TCGA‐GBM (HG‐U133A), Bao‐GBM, Gill‐GBM, LeeY‐GBM, and Oh‐GBM datasets (Figure 2L–S).

Based on the Ivy GAP database, we investigated C1R expression across various anatomical regions of tumors. The results indicated that C1R exhibits specific expression patterns in different anatomical regions of GBM, with significantly higher expression in cellular tumor (CT) regions compared to leading edge (LE) and infiltrating tumor (IT) regions (*p* < 0.001). C1R expression in pseudopalisading cells around necrosis (PAN) was also significantly higher than in leading edge (LE) and infiltrating tumor (IT) regions (*p* < 0.001) (Figure 2T). Reverse phase protein array (RPPA) analysis further revealed upregulation of multiple EMT‐related proteins in the high C1R expression group, including PAI‐1, Caveolin‐1, Collagen‐VI, Fibronectin, and Annexin.1 (Figure 2U).

Furthermore, correlation analysis between C1R protein levels and various tumor microenvironment‐related signatures revealed that C1R positively correlated with multiple critical microenvironment scores, including ESTIMATE Stromal Score, ESTIMATE Immune Score, xcell stroma score, xcell immune score, xcell microenvironment score, xcell Macrophage score, xcell Macrophage M2 score, and xcell B cell score (Figure S1I). These findings indicated that C1R was highly expressed in the mesenchymal subtype of GBM and closely associated with the EMT process, suggesting that C1R may play an important role in mesenchymal transformation and malignant progression of GBM.

### 3.3 TEFT Downregulated C1R and Inhibited Epithelial‐Mesenchymal Transition

**FIGURE 3:**
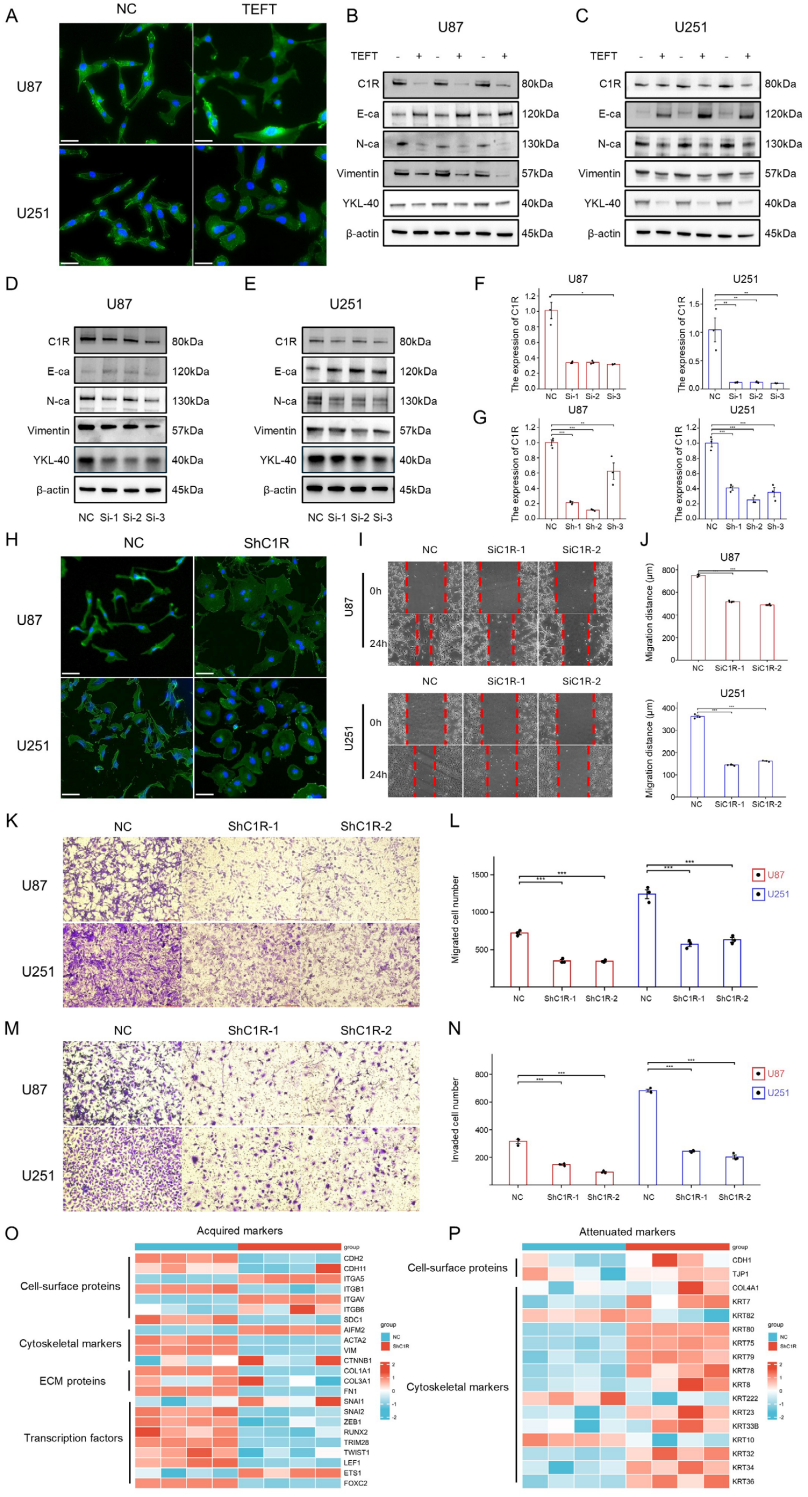
TEFT‐downregulated C1R and inhibited epithelial‐mesenchymal transition. (A) Cell morphology staining of U87 and U251 cells with phalloidin (green) and DAPI (blue) showing morphological changes before and after TEFT treatment. Scale bar = 50 μm. (B, C) Western blot analysis of the expression changes of EMT related markers in U87 and U251 cells after TEFT treatment. (D, E) Western blot analysis of the expression changes of EMT related markers in U87 and U251 cells transfected with NC or C1R‐specific siRNAs (Si‐1, Si‐2, Si‐3). (F, G) RT‐qPCR detection of knockout efficiency of C1R gene in U87 and U251 cell lines. Data are presented as mean ± SD. *p < 0.05, **p < 0.01, ***p < 0.001. (H) Cell morphology staining of U87 and U251 cells with phalloidin (green) and DAPI (blue) showing morphological changes after stable C1R knockdown. Scale bar = 50 μm. (I, J) The wound healing test showed the migration ability of U87 and U251 cells transfected with NC or ShC1R at 0 and 24 h. The red dashed line represents the edge of the wound. (K, L) Downregulation of C1R by siRNA transfection resulted in a decrease in the migratory abilities of U87 and U251 cells as determined by transwell analysis. (M, N) Downregulation of C1R by siRNA transfection resulted in a decrease in the invasive abilities of U87 and U251 cells as determined by transwell analysis. (O, P) Heatmap showing differential expression of EMT‐related markers following transcriptome sequencing of U87 cells with stable C1R knockdown compared to control.

To further investigate the relationship between TEFT and EMT, we observed morphological changes in GBM cells following electric field treatment. After TEFT treatment, U87 and U251 cells exhibited significant morphological alterations, with retraction of cellular projections and transformation from an elongated spindle shape to a rounded shield‐like morphology (Figure 3A). Western blot analysis revealed that TEFT treatment increased expression of the epithelial marker E‐cadherin while decreasing expression of mesenchymal markers N‐cadherin, Vimentin, and YKL‐40 in both U87 and U251 cells, indicating that TEFT treatment suppressed the EMT process in GBM cells (Figure 3B,C).

To explore the role of C1R in EMT, we employed RNA interference technology to knock down C1R expression. RT‐qPCR assessment of knockdown efficiency for three siRNA and shRNA sequences identified Si‐1 and Sh‐2 sequences as having optimal C1R inhibitory effects (Figure 3F,G). Following C1R knockdown, Western blot detection showed upregulation of E‐cadherin and downregulation of N‐cadherin, Vimentin, and YKL‐40, suggesting that C1R inhibition can reverse the EMT process in GBM cells (Figure 3D,E). Cellular morphological staining further confirmed that GBM cells with stable C1R knockdown underwent morphological changes from a mesenchymal phenotype toward a more epithelial‐like phenotype, transforming from spindle‐shaped cells to rounded cells with fewer pseudopodia (Figure 3H).

Functional experiments were conducted to assess the impact of C1R knockdown on cell migration and invasion capabilities of GBM cells. Wound healing assays demonstrated that U87 and U251 cells treated with siC1R‐1 and siC1R‐2 exhibited significantly shorter migration distances after 24 h compared to control groups (Figure 3I,J). Transwell migration assays (Figure 3K,L) and invasion assays (Figure 3M,N) similarly confirmed that knockdown of C1R expression using shC1R‐1 and shC1R‐2 sequences significantly decreased both migratory cell numbers and invasive cell numbers in U87 and U251 cells.

To further elucidate the relationship between C1R and EMT at the transcriptome level, we performed transcriptome sequencing analysis on U87 cells with stable C1R knockdown and their control groups (Figure 3O,P). The heatmaps revealed significant changes in EMT‐related marker expression [44]. In the C1R knockdown group, EMT‐acquired markers such as cell‐surface proteins (CDH2, CDH11, ITGB1), cytoskeletal markers (ACTA2, VIM), ECM proteins (COL1A1, COL3A1, FN1), and transcription factors (SNAI2, ZEB1, RUNX2, FOXC2) showed decreased expression, while EMT‐attenuated markers including cell‐surface proteins (CDH1, TJP1) and cytoskeletal markers (KRT80, KRT75, KRT79) exhibited increased expression. These results emphasized the crucial role of C1R in promoting EMT‐driven malignancy in GBM progression.

### 3.4 TGF‐β/SMAD2/3/STAT3 Was a Potential Upstream Regulatory Pathway of C1R

**FIGURE 4:**
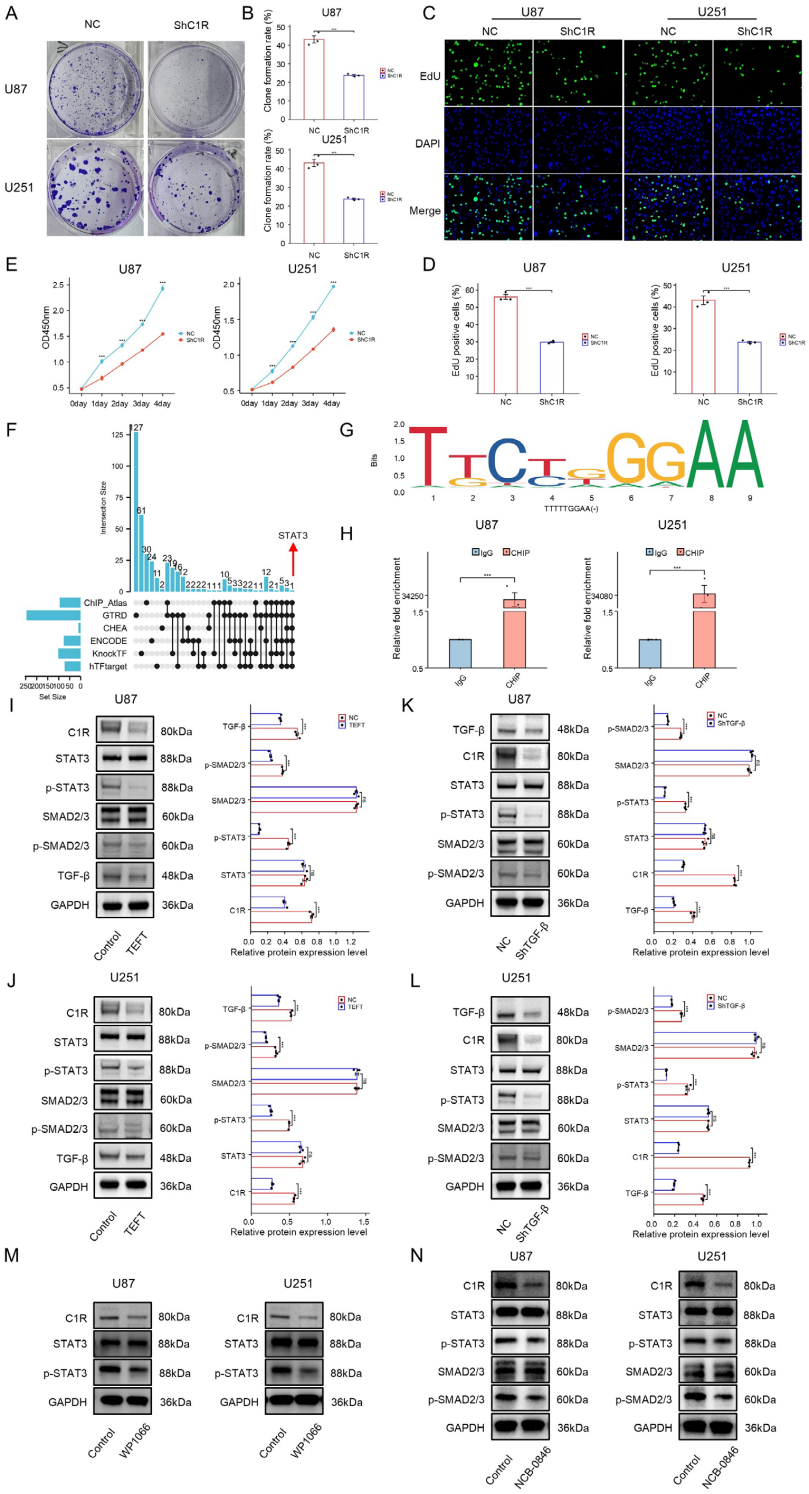
TGF‐β/SMAD2/3/STAT3 was a potential upstream regulatory pathway of C1R. (A, B) Colony formation assays of U87 and U251 cells transfected with ShC1R or NC. Data are mean ± SD, ***p < 0.001, *t*‐test. (C, D) EdU proliferation assay evaluating cell proliferation in U87 and U251 cells transfected with ShC1R or NC. Data are mean ± SD, ***p < 0.001, *t*‐test. (E) CCK‐8 assays showing the proliferation curves of U87 and U251 cells over 4 days following C1R knockdown. Data are mean ± SD, ***p < 0.001, Two‐way ANOVA. (F) Bioinformatics analysis using multiple transcription factor databases (ChIP‐Atlas, GTRD, CHEA, ENCODE, KnockTF, hTFtarget) to predict upstream regulators of C1R. (G) Conserved sequence logo of the STAT3 transcription factor binding site predicted by the JASPAR database, along with the highest‐scoring predicted binding site. (H) ChIP‐qPCR analysis in U87 and U251 cells confirmed that STAT3 functions as a direct transcriptional regulator of C1R (***p < 0.001). (I, J) WB analysis of U87 and U251 cells after TEFT treatment showing decreased the expression of C1R, p‐STAT3, p‐SMAD2/3, and TGF‐β. (K, L) WB analysis of U87 and U251 cells after knockdown TGF‐β showing decreased the expression of C1R, p‐STAT3, and p‐SMAD2/3. Data are mean ± SD; ns, *p* ≥ 0.05; *p < 0.05; **p < 0.01; ***p < 0.001; *t*‐test. (M) WB analysis showing that inhibition of STAT3 phosphorylation with a small molecule inhibitor (WP1066) significantly reduced C1R expression in U87 and U251 cells. (N) WB analysis showing that inhibition of SMAD2/3 phosphorylation with a small molecule inhibitor NCB‐0846 significantly reduced p‐STAT3 and C1R protein expression in U87 and U251 cells.

This study systematically investigated the critical role of C1R in GBM cell proliferation and its upstream molecular regulatory network. Using lentivirus‐mediated RNA interference (ShC1R) to knock down C1R expression, we observed a significant inhibitory effect on GBM cell proliferation. Colony formation assays revealed that, compared to the NC group, the ShC1R group of U87 and U251 cells exhibited a significantly reduced colony formation rate (U87: *p* < 0.001; U251: *p* < 0.001) (Figure 4A,B). To further validate these findings, we performed EdU proliferation assays to evaluate DNA synthesis activity. Fluorescence microscopy observations showed that the EdU positivity rate (green fluorescence) in U87 cells of the ShC1R group was notably lower than in the NC group, with quantitative analysis results demonstrating statistically significant differences (*p* < 0.001). The U251 cell line exhibited consistent trends, with the proportion of EdU‐positive cells in the ShC1R group significantly lower than in the NC group (*p* < 0.001) (Figure 4C,D). CCK‐8 proliferation assays revealed that from Day 0 to Day 4, the OD values of U87 and U251 cells with C1R knockdown remained consistently and significantly lower than those of the NC group (Figure 4E).

To investigate the molecular regulatory mechanisms underlying C1R expression, we conducted multi‐omics comparative analyses to determine which regulatory layer most strongly influences C1R protein abundance. Pan‐cancer analysis revealed that among multiple omics features including protein, mRNA, somatic copy number variations (SCNV), and methylation, mRNA levels exhibited the strongest correlation with C1R protein abundance across various cancer types (Figure S1E), indicating that transcriptional regulation represents the primary mechanism controlling C1R expression. This observation was further validated in GBM, where C1R protein levels demonstrated a robust positive correlation with mRNA levels (Rho = 0.72, *p* = 3.9e‐17) (Figure S1F), confirming that transcriptional control mechanisms play a predominant role in determining C1R protein expression in GBM. These findings prompted us to identify the specific signaling pathways and transcription factors responsible for regulating C1R transcription.

Given the prominent role of C1R in EMT regulation and the well‐established connection between TGF‐β signaling and EMT, we investigated the potential relationship between C1R expression and TGF‐β pathway activity. Multi‐omics pan‐cancer analysis demonstrated strong positive correlations between C1R abundance (at both protein and RNA levels) and TGF‐β signaling pathway activity across multiple cancer types (Figure S1C). In GBM specifically, C1R protein levels exhibited a significant positive correlation with HALLMARK TGF‐β signaling scores (Rho = 0.52, *p* = 4.7e‐8) (Figure S1D), suggesting that TGF‐β signaling may represent a critical upstream regulatory mechanism of C1R expression.

**FIGURE 5:**
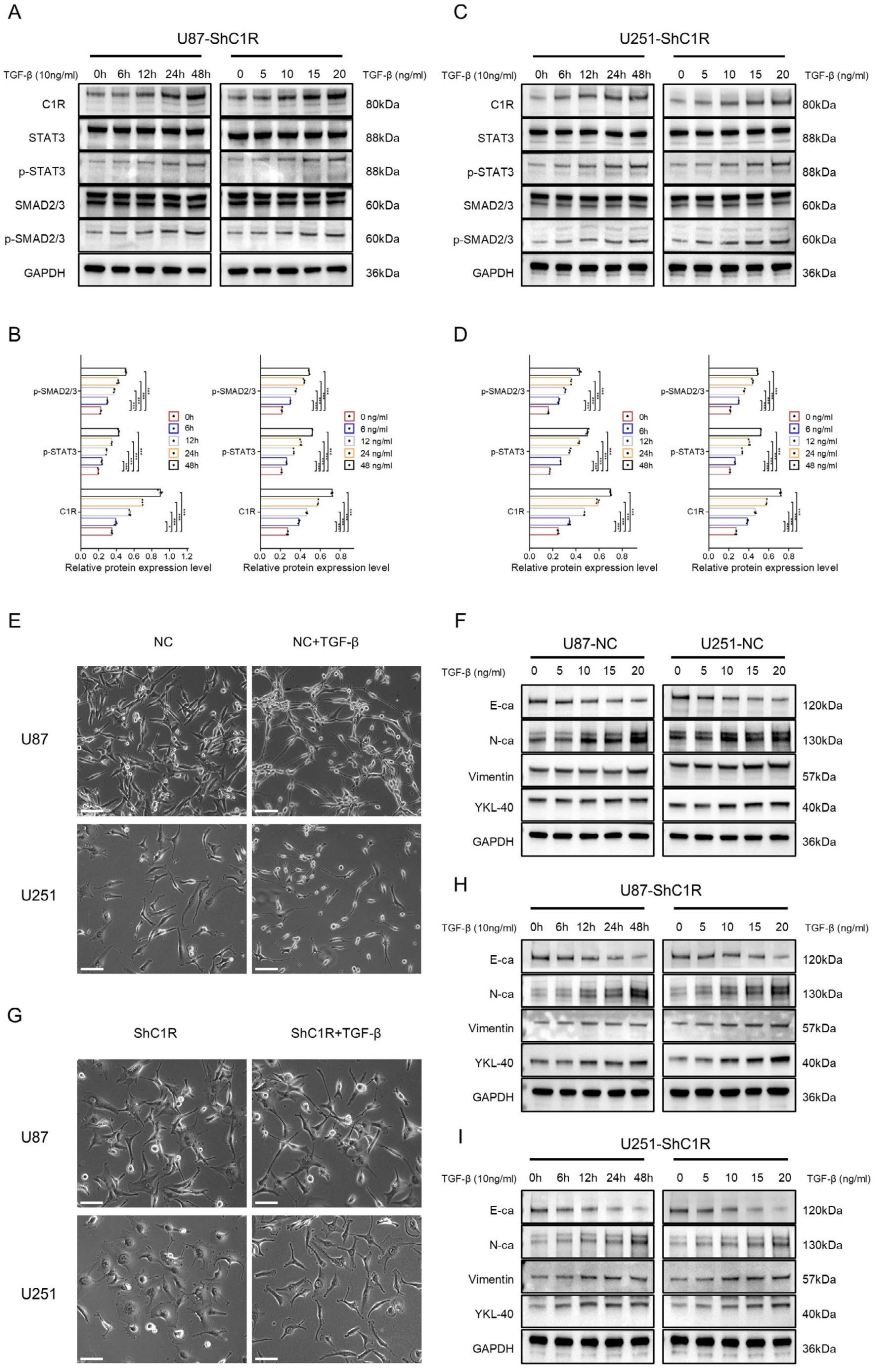
Exogenous TGF‐β restored C1R expression and reversed MET induced by C1R knockdown. (A, B) WB and quantification (*n* = 3) analysis exhibited that the expression levels of C1R, p‐STAT3, and p‐SMAD2/3 significantly increased in a dose‐dependent (0–20 ng/mL) and time‐dependent (0–48 h) manner after TGF‐β treatment in U87‐ShC1R cells. (C, D) WB and quantification (*n* = 3) analysis exhibited that the expression levels of C1R, p‐STAT3, and p‐SMAD2/3 significantly increased in a dose‐dependent (0–20 ng/mL) and time‐dependent (0–48 h) manner after TGF‐β treatment in U251‐ShC1R cells. Data are mean ± SD; ns, *p* ≥ 0.05; *p < 0.05; **p < 0.01; ***p < 0.001; one‐way ANOVA. (E) Morphological changes in normal U87 and U251 cells after treatment with 20 ng/mL TGF‐β for 48 h. Scale bar = 100 μm. (F) WB analysis of EMT markers in U87‐NC and U251‐NC cells treated with different concentrations of TGF‐β (0, 5, 10, 15, and 20 ng/mL). (G) Morphological changes in U87‐ShC1R and U251‐ShC1R cells after treatment with 20 ng/mL TGF‐β for 48 h. Scale bar = 100 μm. (H, I) WB exhibited that the expression levels of mesenchymal markers (N‐cadherin, Vimentin, YKL‐40) significantly increased while epithelial marker (E‐cadherin) decreased in a dose‐dependent (0–20 ng/mL) and time‐dependent (0–48 h) manner after TGF‐β treatment in U87‐ShC1R and U251‐ShC1R cells.

To explore the upstream regulatory mechanisms of C1R, we conducted bioinformatics analysis using multiple transcription factor databases, including ChIP‐Atlas, GTRD, CHEA, ENCODE, KnockTF, and hTFtarget. The results identified STAT3 as the only common predicted transcription factor across these databases, suggesting that STAT3 may function as a key upstream regulator of C1R (Figure 4F). JASPAR database analysis identified a conserved STAT3 binding motif within the C1R promoter sequence, with TTTTTGGAA representing the highest‐scoring predicted binding site (Figure 5G). Subsequent ChIP‐qPCR experiments in U87 and U251 cells validated direct binding of STAT3 to the C1R promoter, confirming its role as a transcriptional regulator of C1R (*p* < 0.001) (Figure 5H). Western blot analysis demonstrated that following TEFT treatment, C1R (U87: ***p < 0.001; U251: ***p < 0.001) and TGF‐β (U87: ***p < 0.001; U251: ***p < 0.001) expression levels in U87 and U251 cells significantly decreased, accompanied by reduced phosphorylation levels of STAT3 (U87: ***p < 0.001; U251: ***p < 0.001) and SMAD2/3 (U87: ***p < 0.001; U251: ***p < 0.001), while total STAT3 and SMAD2/3 protein levels remained relatively unchanged (Figure 4I,J). Furthermore, when TGF‐β expression was knocked down using shRNA (ShTGF‐β), C1R expression levels in U87 and U251 cells similarly decreased, accompanied by reduced phosphorylation levels of STAT3 and SMAD2/3 (Figure 4K,L).

To further validate the role of STAT3 in regulating C1R expression, we employed the small molecule inhibitor WP1066 to specifically inhibit STAT3 phosphorylation. Western blot analysis revealed that WP1066 treatment significantly reduced C1R expression levels in both U87 and U251 cells, confirming that STAT3 phosphorylation is essential for maintaining C1R expression (Figure 4M). Moreover, inhibition of SMAD2/3 phosphorylation using NCB‐0846 resulted in concurrent reduction of both p‐STAT3 and C1R protein levels in U87 and U251 cells (Figure 4N). These pharmacological intervention studies provide compelling evidence that the TGF‐β/SMAD2/3/STAT3 signaling pathway represents a critical upstream regulatory mechanism regulating C1R expression in GBM cells.

### 3.5 Exogenous TGF‐β Restored C1R Expression and Reversed MET Induced by C1R Knockdown

To further validate the regulatory role of the TGF‐β signaling pathway on C1R expression and its function in the EMT process of GBM cells, we designed exogenous TGF‐β addition experiments, conducting both dose‐dependent and time‐dependent TGF‐β stimulation experiments on GBM cells. When U87‐ShC1R and U251‐ShC1R cells were treated with different concentrations (0, 5, 10, 15, and 20 ng/mL) of TGF‐β for 48 h, Western blot analysis revealed that C1R expression levels increased with rising TGF‐β concentrations. This increase was accompanied by enhanced phosphorylation levels of STAT3 and SMAD2/3 (Figure 5A–D). Similarly, in U87‐ShC1R and U251‐ShC1R cells, treatment with 10 ng/mL TGF‐β for varying durations (0, 6, 12, 24, and 48 h) exhibited comparable trends, with C1R expression upregulation and gradual increases in STAT3 and SMAD2/3 phosphorylation levels as treatment duration extended, indicating that TGF‐β activated the SMAD2/3/STAT3/C1R signaling pathway in a concentration‐ and time‐dependent manner (Figure 5A–D).

Morphological observations showed that normal U87 and U251 cells developed elongated cellular projections and more pronounced mesenchymal features after treatment with 20 ng/mL TGF‐β for 48 h (Figure 5E). Western blot analysis confirmed that mesenchymal markers such as N‐cadherin, YKL‐40, and Vimentin were upregulated in normal U87 and U251 cells following treatment with increasing concentrations of TGF‐β (0, 5, 10, 15, and 20 ng/mL) for 48 h. Concurrently, epithelial marker E‐cadherin expression decreased progressively with rising TGF‐β concentrations (Figure 5F). Additionally, when U87‐ShC1R and U251‐ShC1R cells were treated with exogenous TGF‐β (20 ng/mL) for 48 h, these cells similarly transitioned from epithelial‐like morphology to mesenchymal‐like morphology. This reversal indicated that supplementing TGF‐β can reverse the MET process induced by C1R knockdown (Figure 5G). Western blot analysis further verified that in U87‐ShC1R and U251‐ShC1R cells treated with different concentrations (0, 5, 10, 15, and 20 ng/mL) of TGF‐β for 48 h, expression levels of mesenchymal markers N‐cadherin, YKL‐40, and Vimentin increased with rising concentrations, while expression levels of the epithelial marker E‐cadherin showed a decreasing trend (Figure 5H,I). Similarly, for treatment with 10 ng/mL TGF‐β at different time points (0, 6, 12, 24, and 48 h), expression changes in EMT‐related markers also exhibited time dependency. These results indicated that TGF‐β supplementation can activate the SMAD2/3/STAT3/C1R signaling pathway to reverse the MET process induced by C1R knockdown. This activation promoted a more invasive mesenchymal‐like phenotype in GBM cells, thereby enhancing the malignancy of GBM.

### 3.6 In Vivo Studies Validate the Inhibitory Effect of TEFT on TGF‐β/C1R Axis and EMT

**FIGURE 6:**
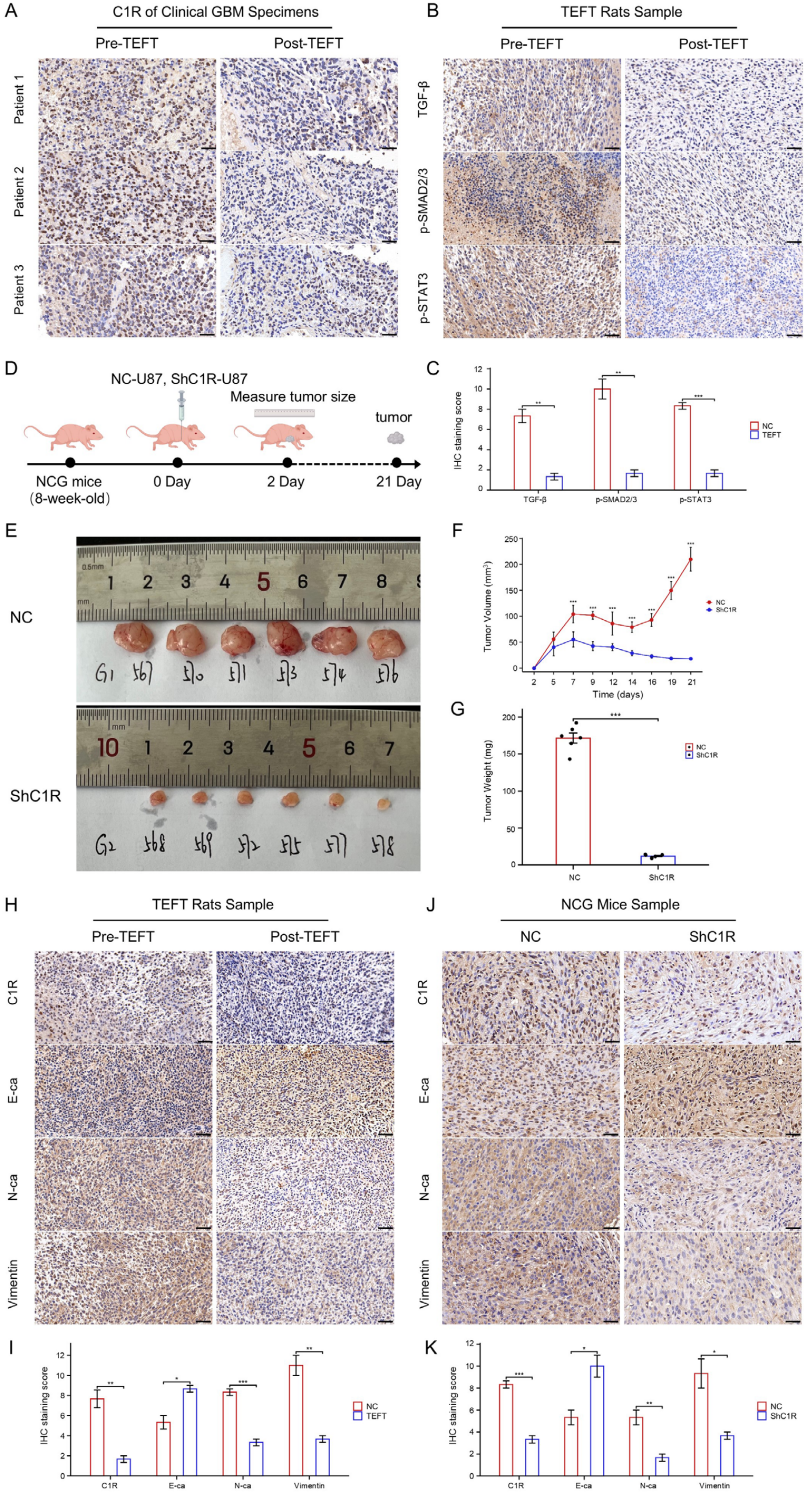
In vivo studies validate the inhibitory effect of TEFT on the TGF‐β/C1R axis and EMT. (A) Immunohistochemical staining of C1R in clinical GBM specimens from three patients before and after TEFT treatment. Scale bar = 50 μm. (B) Immunohistochemical staining of TGF‐β, p‐SMAD2/3, and p‐STAT3 in rat GBM samples before and after TEFT treatment. Scale bar = 50 μm. (C) Quantification of IHC staining scores for TGF‐β, p‐SMAD2/3, and p‐STAT3 in rat GBM samples before and after TEFT treatment. Data are mean ± SD, **p < 0.01, ***p < 0.001, *t*‐test. (D) Schematic diagram of the NCG mice subcutaneous tumor model experiment. (E) Representative images of subcutaneous tumors from NC and ShC1R groups at Day 21 postinjection. (F) Growth curves of tumor volume in NC and ShC1R groups over 21 days. Data are mean ± SD, ***p < 0.001, Two‐way ANOVA. (G) Quantification of tumor weight in NC and ShC1R groups at Day 21 postinjection. Data are mean ± SD. ***p < 0.001, *t*‐test. (H, I) Immunohistochemical analysis of C1R, E‐cadherin, N‐cadherin, and Vimentin in rat GBM samples before and after TEFT treatment. (J, K) Immunohistochemical analysis of C1R, E‐cadherin, N‐cadherin, and Vimentin in subcutaneous tumors from NC and ShC1R groups. Scale bar = 50 μm. Data are mean ± SD, *p < 0.05, **p < 0.01, ***p < 0.001, *t*‐test.

Based on our previous in vitro findings, we further validated the inhibitory effect of TEFT on GBM malignant progression in clinical samples and animal models. Immunohistochemical analysis of GBM tissue from three patients who underwent TEFT treatment revealed a significant reduction in C1R expression posttreatment (Figure 6A). Immunohistochemical analysis of rat brain GBM samples demonstrated that TEFT treatment markedly reduced the expression levels of TGF‐β (**p < 0.01), p‐SMAD2/3 (**p < 0.01) and p‐STAT3 (***p < 0.001), suggesting that TEFT effectively suppressed the TGF‐β signaling pathway (Figure 6B,C).

Additionally, we established a subcutaneous transplant tumor model using NCG immunodeficient mice, with the experimental procedure as shown in Figure 6D. Tumors formed by U87‐ShC1R cells were notably smaller than those formed by U87‐NC cells after 21 days (Figure 6E). Tumor volume growth curves showed that C1R knockdown significantly slowed the rate of tumor volume increase (***p < 0.001) (Figure 6F), ultimately resulting in markedly reduced final tumor weights (***p < 0.001) (Figure 6G).

In rat brain GBM samples treated with TEFT, immunohistochemical analysis showed decreased C1R (*p < 0.05) expression accompanied by increased expression of the epithelial marker E‐cadherin (*p < 0.05) and decreased expression of mesenchymal markers N‐cadherin (***p < 0.001) and Vimentin (**p < 0.01) (Figure 6H,I). Similarly, in the NCG mouse tumor model, immunohistochemical analysis of the ShC1R group compared to the control group showed decreased C1R (***p < 0.001) expression, elevated expression of the epithelial marker E‐cadherin (*p < 0.05), and reduced expression of the mesenchymal markers N‐cadherin (**p < 0.01) and Vimentin (*p < 0.05) (Figure 6J,K). These findings collectively demonstrate that TEFT inhibits GBM growth and EMT through suppression of the TGF‐β signaling pathway and downregulation of C1R.

## 4 Discussion

TEFT represents a groundbreaking advancement in GBM treatment, standing as the only novel therapy approved and incorporated into National Comprehensive Cancer Network (NCCN) guidelines over the past decade [45, 46]. Despite its demonstrated clinical efficacy in extending both OS and PFS when combined with temozolomide, the precise molecular mechanisms and critical targets mediating TEFT's anti‐GBM effects remain incompletely understood [10, 47]. In this study, we have identified the complement component C1R as a key molecular target of TEFT's therapeutic effects in GBM. Our findings reveal that TEFT inhibits the TGF‐β/SMAD2/3/STAT3 signaling pathway, which subsequently downregulates C1R expression, ultimately suppressing EMT and reducing the aggressive mesenchymal phenotype of GBM. This mechanistic insight not only enhances our understanding of TEFT's mode of action but also identifies potential biomarkers and therapeutic targets for improving GBM treatment strategies.

EMT is a critical biological process in which epithelial cells lose polarity and adhesion properties while acquiring mesenchymal characteristics with enhanced migration and invasion capabilities [48]. EMT plays a key role in tumor development, promoting metastasis, treatment resistance, and immune evasion [49]. Several studies suggest that glioma cells undergo EMT‐like processes during progression to higher grades [50, 51]. Unlike tumors originating from epithelial tissues, GBM derives from glial cells and rarely metastasizes distantly, yet its local infiltration and recurrence capabilities are closely linked to EMT‐like processes [52, 53]. Among GBM molecular subtypes, the MES subtype exhibits the most aggressive phenotype, characterized by enhanced invasiveness, rapid recurrence, and significant resistance to conventional radiotherapy and chemotherapy [54, 55, 56]. In GBM, MES‐like malignant cells display high levels of EMT‐related markers and express genes involved in extracellular matrix remodeling, inflammation, and cell motility [57]. Our single‐cell sequencing analysis revealed that C1R is predominantly expressed in MES‐like malignant cells and enriched in regions with high HALLMARK EMT scores. Additionally, across multiple GBM cohorts, C1R expression was significantly higher in the MES subtype compared to PN and CL subtypes. The marked C1R upregulation in PNA observed in our Ivy GAP analysis aligns with recent spatial transcriptomic evidence that perinecrotic regions constitute severely hypoxic, immunosuppressive niches where HIF‐1α activation drives mesenchymal transition and enhances TGF‐β signaling [58, 59, 60]. This spatial enrichment of C1R in these microenvironments reflects the convergence of hypoxia‐driven and TGF‐β‐mediated pathways that collectively sustain aggressive mesenchymal phenotypes in GBM. These findings establish the first connection between C1R and mesenchymal characteristics in GBM, suggesting C1R may play a crucial role in the EMT process of GBM and highlighting its potential as a biomarker for mesenchymal GBM and an important target for therapeutic intervention.

C1R is a key component of the C1 complex involved in classical complement activation and antigen presentation [61]. C1R plays an important role in immune regulation and tumor progression [62]. Zhang et al. [63] found in pancreatic cancer that neoadjuvant therapy can induce upregulation of complement components, including C1R, in the TME, thereby alleviating immune cell exhaustion and enhancing its response to immunotherapy. Xiao et al. [30] discovered in glioma research that C1R is associated with immune cell infiltration and may drive the hypoxic phenotype of perinecrotic GBM, thereby affecting hypoxia‐induced glioma stemness. Moreover, recent studies have identified antitumor effects of C1R beyond its role in the complement system. Riihilä et al. [33] found that tumor cell‐derived complement component C1R can promote malignant progression of cutaneous squamous cell carcinoma by activating ERK1/2 and PI3K signaling pathways. Their subsequent research further revealed that C1R can increase the invasiveness of cutaneous squamous cell carcinoma by promoting the expression of invasion‐associated matrix metalloproteinases (MMPs) [34]. Ma et al. [35] demonstrated that C1R inhibits invasive behavior of hepatocellular carcinoma in vitro and in vivo by suppressing HIF‐1α‐regulated glycolysis. These studies indicate that C1R not only functions in the complement system but also participates in tumor initiation and progression through multiple mechanisms. Given C1R's established role in complement activation and immune modulation, our findings suggest that TEFT‐mediated C1R downregulation may have broader implications for tumor immunology, potentially influencing immune cell infiltration and reshaping the immunosuppressive microenvironment characteristic of mesenchymal GBM.

In our research, we discovered for the first time that downregulation of C1R following TEFT contributes to inhibition of EMT in GBM, with subsequent mechanistic studies revealing the involvement of the TGF‐β signaling pathway in this critical process. The TGF‐β signaling pathway is widely recognized as an important mechanism regulating EMT in various cancers [64], promoting mesenchymal transition by activating downstream effector molecules such as SMAD2 and SMAD3, which coordinate a series of transcriptional programs driving tumor cell migration, invasion, and immune evasion [65, 66]. Our research found that TEFT inhibits the TGF‐β signaling pathway, reducing phosphorylation of SMAD2/3 and STAT3, thereby suppressing C1R expression and resulting in weakened EMT and invasive capabilities of GBM. Stimulating C1R‐knockdown GBM cells with TGF‐β growth factor could restore their mesenchymal characteristics. Furthermore, in vitro and in vivo experiments revealed the inhibitory effect of C1R knockdown on GBM growth. Accumulating evidence has demonstrated that complement system components play critical roles in regulating EMT across various malignancies [67, 68, 69]. While our results demonstrate that C1R knockdown alters E‐cadherin and Vimentin expression, the precise regulatory mechanism warrants further investigation. It remains to be determined whether C1R directly modulates EMT markers or functions indirectly through master EMT transcription factors such as Snail, Slug, or ZEB1/2. These findings suggest that C1R may be a novel therapeutic target for GBM, and inhibiting C1R and modulating TGFβ signaling may reverse EMT and enhance the efficacy of TEFT.

According to reports, TEFT combined with other therapeutic approaches has shown promising efficacy in various cancer models. Giladi et al. [70] found in non‐small cell lung cancer research that TEFT combined with pemetrexed, cisplatin, or paclitaxel significantly enhanced therapeutic effects compared to monotherapy. Jang et al. [71] reported synergistic effects when TEFT was combined with sorafenib, with TEFT enhancing the sensitivity of hepatocellular carcinoma cell lines to sorafenib. Kim et al. [72] also reported that TEFT enhanced sensitivity of GBM xenograft models to sorafenib treatment in nude mice, and sorafenib increased the susceptibility of GBM cells to TEFT‐induced apoptosis through inhibition of STAT3. Results from a phase III clinical trial for newly diagnosed GBM showed that TEFT combined with TMZ significantly improved PFS and overall OS compared to TMZ alone, without affecting health‐related quality of life (HRQoL) [10, 73]. Our research is the first to reveal the mechanism by which TEFT downregulates the TGF‐β/SMAD2/3/STAT3 signaling pathway to inhibit C1R expression and reverse EMT, providing a molecular basis for the antitumor effects of TEFT. Both in vitro and in vivo experiments confirmed that either TEFT treatment or C1R knockdown effectively inhibits malignant progression of GBM, with potential efficacy against the mesenchymal subtype. Therefore, our findings emphasize the potential for developing combination therapies targeting TEFT and the TGF‐β/C1R axis to improve GBM treatment efficacy. Moreover, intraoperative local electric field application to residual tumor tissue may represent a promising strategy to maximize TEFT efficacy, particularly given the marked elevation of C1R expression in MES‐subtype tumor cores and perinecrotic regions. These anatomical zones may serve as priority targets for personalized intraoperative electrode placement, enabling electric field configurations tailored to individual tumor molecular profiles. For patients with highly invasive and treatment‐resistant mesenchymal subtype GBM, combining TEFT with TGF‐β/C1R targeted inhibitors may synergistically suppress mesenchymal transition, enhancing treatment sensitivity and efficacy.

Several limitations of this study need to be addressed in future research. First, although we confirmed the role of C1R in GBM across multiple datasets and cell lines, the high heterogeneity of GBM suggests significant variations among different tumor subtypes and individual patients, potentially affecting the universality of TEFT treatment and TGF‐β/C1R targeted therapy. Second, our in vivo experiments primarily relied on subcutaneous xenograft models, lacking validation in intracranial orthotopic xenograft models, which cannot fully simulate the complex growth environment of GBM in the brain. Additionally, while we revealed the upstream mechanism of TGF‐β/SMAD2/3/STAT3 pathway regulation of C1R expression, the downstream molecular mechanisms by which C1R mediates EMT have not been fully elucidated. Future research needs to validate these findings in models that better reflect the biological characteristics of GBM and further explore the downstream regulatory network of C1R to develop more precise combination therapeutic strategies involving TEFT and targeted therapies.

## 5 Conclusion

TEFT inhibits GBM progression by suppressing the TGF‐β/SMAD2/3/STAT3/C1R axis, thereby attenuating EMT and reducing tumor aggressiveness. Our study identifies C1R as a key molecular target primarily expressed in mesenchymal‐like malignant cells, where it promotes invasiveness and treatment resistance. Knockdown of C1R significantly reduces tumor growth and reverses mesenchymal characteristics in GBM cells, while exogenous TGF‐β restores C1R expression and the mesenchymal phenotype. These findings uncover a novel mechanism of TEFT action and establish C1R as a potential biomarker and therapeutic target for GBM, particularly for developing combination strategies to overcome treatment resistance in aggressive mesenchymal subtypes.

## Author Contributions

J.C. wrote the manuscript. J.C., Y.L., and Q.L. conducted bioinformatics data analysis and acquired experiment data. All authors discussed the results. H.L., C.S., X.C., X.Z., J.L., Y.F., L.Q., and J.L. contributed to validation and revised the manuscript. Z.L. and L.C. reviewed, supervised, and acquired funding support. All authors have read and approved the final manuscript.

## Funding

This work was supported by National Natural Science Foundation of China (82172680, 82373220, 82473264, and 82403942).

## Ethics Statement

The study was approved by the Ethics Committee of People's Liberation Army General Hospital, with the approval number S2018‐089‐01.

## Consent

The authors have nothing to report.

## Conflicts of Interest

The authors declare no conflicts of interest. Ling Chen is an Academic Editor of *CNS Neuroscience and Therapeutics* and a coauthor of this article. To minimize bias, they were excluded from all editorial decision‐making related to the acceptance of this article for publication.

## Supporting information


**Figure S1:** Multi‐omics analysis of C1R expression and its associations with EMT, TGF‐β signaling, and tumor microenvironment.

**Table S1:** Primers used in this study.
**Table S2:** The small interfering RNA targeting C1R for transient transfection in this study.
**Table S3:** The short hairpin RNA targeting C1R for lentiviral construction in this study.
**Table S4:** Antibodies used in this study.

## Data Availability

The datasets used and analyzed during the current study are available from the corresponding author on reasonable request.
