# Supplementary figures and images for: Tumor Electric Field Therapy Inhibits TGF‐β/C1R Signaling Axis‐Driven Epithelial‐Mesenchymal Transition in Glioblastoma
Source: CNS Neurosci Ther. 2026 Jan 5;32(1):e70738. doi: 10.1002/cns.70738 (PMC12766902; doi:10.1002/cns.70738)

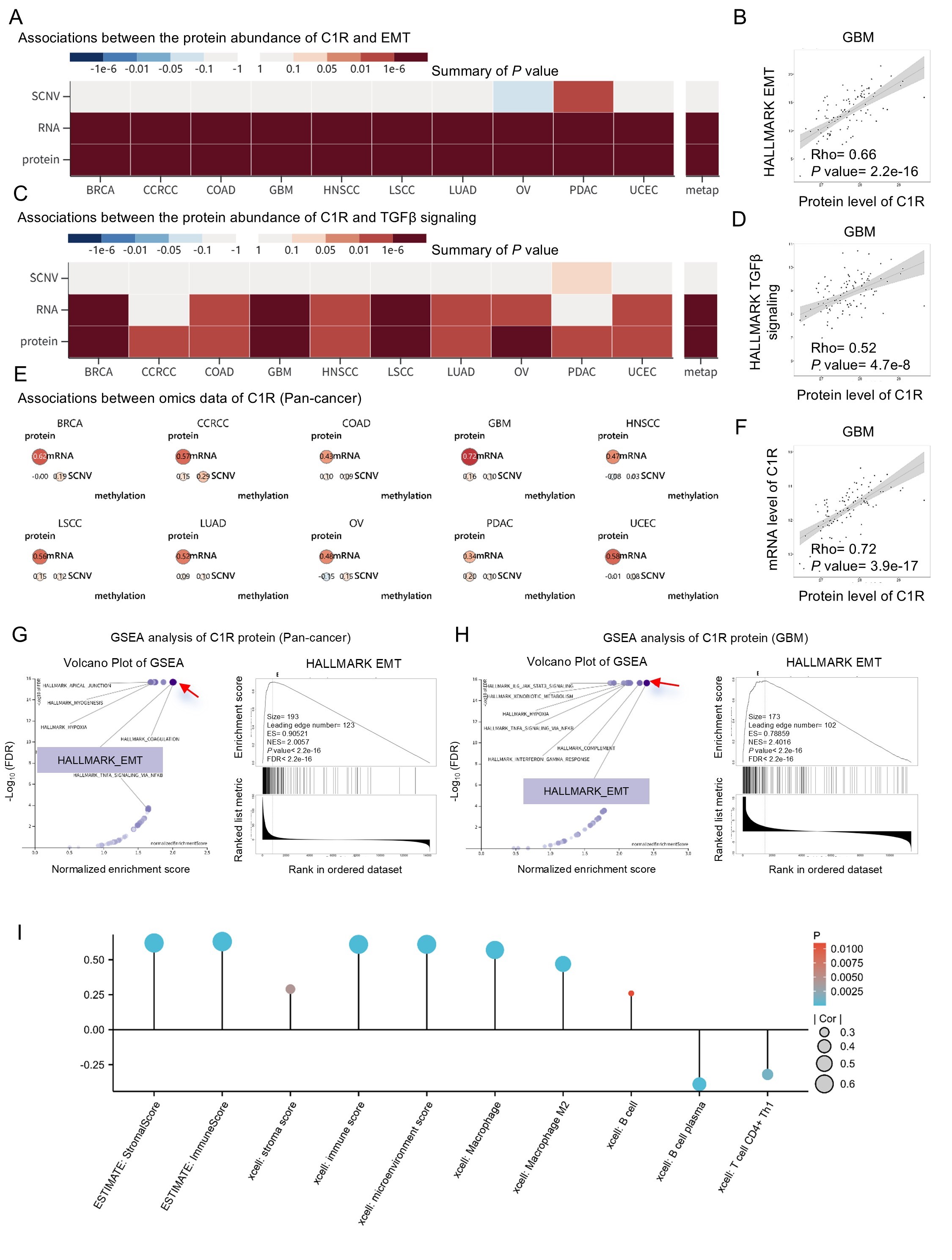

Supplement: Supplementary file 1 — Figure S1: Multi‐omics analysis of C1R expression and its associations with EMT, TGF‐β signaling, and tumor microenvironment. [file CNS-32-e70738-s001.jpg]
