# Supplementary material for: Tumor Electric Field Therapy Inhibits TGF‐β/C1R Signaling Axis‐Driven Epithelial‐Mesenchymal Transition in Glioblastoma
Source: CNS Neurosci Ther. 2026 Jan 5;32(1):e70738. doi: 10.1002/cns.70738 (PMC12766902; doi:10.1002/cns.70738)
Supplement: Supplementary file 2 — Table S1: Primers used in this study. Table S2: The small interfering RNA targeting C1R for transient transfection in this study. Table S3: The short hairpin RNA targeting C1R for lentiviral construction in this study. Table S4: Antibodies used in this study. [file CNS-32-e70738-s002.docx]

**Table S1. Primers used in this study**

| **Gene** | **Forward sequence** **(5' – 3')** | **Reverse sequence (5' – 3')** |
| --- | --- | --- |
| RT-qPCR primers | | |
| C1R | TTCCCCAAGCCTTACCCCAA | GCTGGAAGACGAGCTTCACC |
| Actin | CCTCACCCTGAAGTACCC | AGCCTGGATAGCAACGTACATG |

**Table S2. The Small interfering RNA targeting C1R for transient transfection in this study**

| SiC1R -1 | CAACCACUGUGAUCACAGU (5' – 3') |
| --- | --- |
| SiC1R -2 | GGAGCCUUUUGAUAUUGAU (5' – 3') |
| SiC1R -3 | GGACCAUCAUGUUCUACAA (5' – 3') |

**Table S3. The Short hairpin RNA targeting C1R for lentiviral construction in this study**

| ShC1R -1 | GCGCTACACCACCGAGATCATCTCGAGATGATCTCGGTGGTGTAGCGCTTTTTT |
| --- | --- |
| ShC1R -2 | CTACTGCCATGAGCCATATTACTCGAGTAATATGGCTCATGGCAGTAGTTTTTT |
| ShC1R -3 | CCCGGGAAAGAAGGAATTTATCTCGAGATAAATTCCTTCTTTCCCGGGTTTTTT |

**Table S4. Antibodies used in this study**

| Antibody | Application | Host | Supplier |
| --- | --- | --- | --- |
| C1R | WB and IHC | Rabbit | Cat:# ab185212, Abcam |
| E-cadherin | WB | Rabbit | Cat:# 20874-1-AP, Proteintech |
| N-cadherin | WB | Rabbit | Cat:# 22018-1-AP, Proteintech |
| Vimentin | WB | Rabbit | Cat:# 10366-1-AP, Proteintech |
| YKL-40 | WB | Rabbit | Cat:# 47066, Cell Signaling Technology |
| TGF-β | WB | Rabbit | Cat:# 3709, Cell Signaling Technology |
| STAT3 | WB | Rabbit | Cat:# 51076-2-AP, Proteintech |
| p-STAT3 | WB | Rabbit | Cat:# 9131, Cell Signaling Technology |
| Smad2/3 | WB | Rabbit | Cat:# 8685, Cell Signaling Technology |
| p-Smad2/3 | WB | Rabbit | Cat:# 8828, Cell Signaling Technology |
| GAPDH | WB | Rabbit | Cat:# 10494-1-AP, Proteintech |
| β-actin | WB | Rabbit | Cat:# 4970, Cell Signaling Technology |
| E-cadherin | IHC | Rabbit | Cat:# AF0131, Affinity Biosciences |
| N-cadherin | IHC | Rabbit | Cat:# AF5239, Affinity Biosciences |
| Vimentin | IHC | Rabbit | Cat:# AF7013, Affinity Biosciences |

**Figure S1. Multi-omics analysis of C1R expression and its associations with EMT, TGFβ signaling, and tumor microenvironment.** (A) Heatmap showing associations between C1R expression (protein, RNA, and SCNV levels) and EMT signatures across pan-cancer cohorts (BRCA, CCRCC, COAD, GBM, HNSCC, LSCC, LUAD, OV, PDAC, UCEC). (B) Correlation between C1R protein levels and HALLMARK EMT scores in GBM. (C) Heatmap showing associations between C1R expression (protein, RNA, and SCNV levels) and TGFβ signaling pathway activity across pan-cancer cohorts. (D) Correlation between C1R protein levels and HALLMARK TGFβ signaling scores in GBM. (E) Comparative analysis of associations between C1R protein abundance and multi-omics features (protein, mRNA, SCNV, and methylation) across pan-cancer datasets. (F) Correlation between C1R protein and mRNA levels in GBM. (G) GSEA of C1R protein expression in pan-cancer cohorts. Volcano plot (left) and enrichment plot (right) demonstrated significant enrichment of HALLMARK EMT pathway (Size = 193, Leading edge number = 123, NES = 2.0027, *p* < 2e-10). (H) GSEA of C1R protein expression in GBM. Volcano plot (left) and enrichment plot (right) demonstrated significant enrichment of HALLMARK EMT pathway (Size = 173, Leading edge number = 102, NES = 2.4016, *p* < 2e-10). (I) Correlation analysis between C1R protein levels and tumor microenvironment-related scores.
